# Deaths in the City of Buffalo, for the Month of June, 1864

**Published:** 1864-08

**Authors:** Sandford Eastman

**Affiliations:** Health Physician


*Deaths in the City of Buffalo, for the month of June*, 1864.

Causes of death—Accident [illegible], do. by drowning 6, albuminuria 1, anemia 1, bronchitis 5, liver 1, womb 1, cholera infantum 2, cholera morbus 1, coma 1, consumption 15, convulsons 6, croup 4, croup diphtheritie 1, debility 2, delirium tremens 1, dentition 1, diarrhœa 1, disease of the brain 2, diphtheria 2, erysipelas 1, fever remittent 1, do. puerperal 1, do. scarlet 10, do. tyhhoid 5, do. typhus 1, Inflammation of bowels 3, do. brain 1, do. brain and meninges 3, do, lungs 8, do. womb 1, jaundice 1, marasmus 2, measles 6, old age 1, pyæmia 1, small pox 2, tabes nascentium 1 trismus nascentium 1, unknown 3. Deaths from diseases 116. Still-born 2. The number of deaths in the city in June, 1863, 125 ; average of each June for the five years, 1859 to 1863, inclusive, 112.

Sex—Males 67. Females 51. Total 118. Nativities—United States 86, German States 12. Ireland 9, England 5, Canada 3, Nova Scotia 1, Wales 1, unknown 1. Locality—City at large 95; Hospital Sisters of Charity 6; Catholic Foundling Asylum 6; Erie County Alms House 8; Small Pox Hospital 1; Erie County Penitentiary 1; Providence Insane Asylum 1. Under 1 year 20; under 5 years 36; over 5 years 82. Certified by regular physicians at public institutions 22; by regular physicians in city at large 49; by irregular practitioners 20; by coroner 14; by undertakers 13.

The number of deaths in the first six months of the present year, is 99 more than in the corresponding period of last year; and 59 more than the average for five years.

SANDFORD EASTMAN, M. D., Health Physician.

